# The Prognostic Prediction Value of Positive Lymph Nodes Numbers for the Hypopharyngeal Squamous Cell Carcinoma

**DOI:** 10.3389/fmed.2022.898483

**Published:** 2022-07-04

**Authors:** Wendu Pang, Yaxin Luo, Junhong Li, Danni Cheng, Yufang Rao, Minzi Mao, Ke Qiu, Yijun Dong, Jun Liu, Jian Zou, Haiyang Wang, Fei Chen

**Affiliations:** ^1^Department of Otolaryngology Head and Neck Surgery, West China Hospital, Sichuan University, Chengdu, China; ^2^Department of Epidemiology and Biostatistics, West China School of Public Health and West China Fourth Hospital, Sichuan University, Chengdu, China

**Keywords:** hypopharyngeal squamous cell carcinoma, positive lymph nodes number, prognosis, prediction models, survival predictive values

## Abstract

**Background:**

The current American Joint Committee on Cancer (AJCC) system only considered the importance of the size and laterality of lymph nodes while not the positive lymph node number (PLNN) for hypopharyngeal squamous cell carcinoma (HPSCC).

**Methods:**

A total of 973 patients with HPSCC from the Surveillance, Epidemiology, and End Results database (2004–2015) were identified. Univariate and multivariate Cox regression analyses were used to evaluate the prognostic effects. We applied six Cox regression models to compare the survival prognostic values of PLNN and AJCC systems.

**Results:**

Positive lymph node number showed a significant association with overall survival (OS) and cancer-specific survival (CSS) (*P* < 0.001) in univariate and multivariable analyses. The increased PLNN of HPSCC gave rise to poor OS and CSS. The survival model incorporating a composite of PLNN and TNM classification (C-index for OS:0.682, C-index for CSS:0.702) performed better than other models.

**Conclusions:**

A positive lymph node number could serve as a survival predictor for patients with HPSCC and a complement to enhance the prognostic assessment effects of TNM cancer staging systems.

## Background

Hypopharyngeal squamous cell carcinoma (HPSCC) is one of the most malignant head and neck squamous cell carcinomas (HNSCC), accounting for 5–15% of HNSCC, with a 5-year survival rate of <40% (1). As early symptoms are relatively insidious, tumors often progress to advanced stages when discovered (2). Lymph node metastasis (LNM) is closely related to poor prognosis (3). However, the current American Joint Committee on Cancer (AJCC) staging system only placed weight on the size and laterality of lymph nodes (LNs) for HPSCC, while not considering positive lymph node number (PLNN). Several studies have suggested that PLNN is a clinicopathological risk factor and potential prognostic determinant for patients with thyroid cancer (4), nasopharyngeal carcinoma (5), and oral cavity squamous cell carcinoma (6), while quantification of the prognostic effects of PLNN has not been adequately performed by a substantial HPSCC cohort.

Therefore, we hypothesized that PLNN could serve as a supplement to the AJCC tumor, lymph node, and metastasis (TNM) staging system of HPSCC to assist in better treatment guidance. In this study, we investigated the prognostic effect of PLNN on patients with HPSCC, developed several survival prediction models based on PLNN, and compared their prognostic prediction values to the 6th and 7th AJCC cancer staging systems.

## Methods

### Database Information

Patient data between 2004 and 2015 (year of diagnosis) were obtained from the Surveillance, Epidemiology, and End Results (SEER) database, an authoritative data source of the National Cancer Institute (7) *via* the SEER Stat software (https://seer.cancer.gov). This study was approved by our institutional review board (No. 2019.357), and patient consent was not applicable as SEER data are publicly available.

### Patient Selection

**Figure 1:**
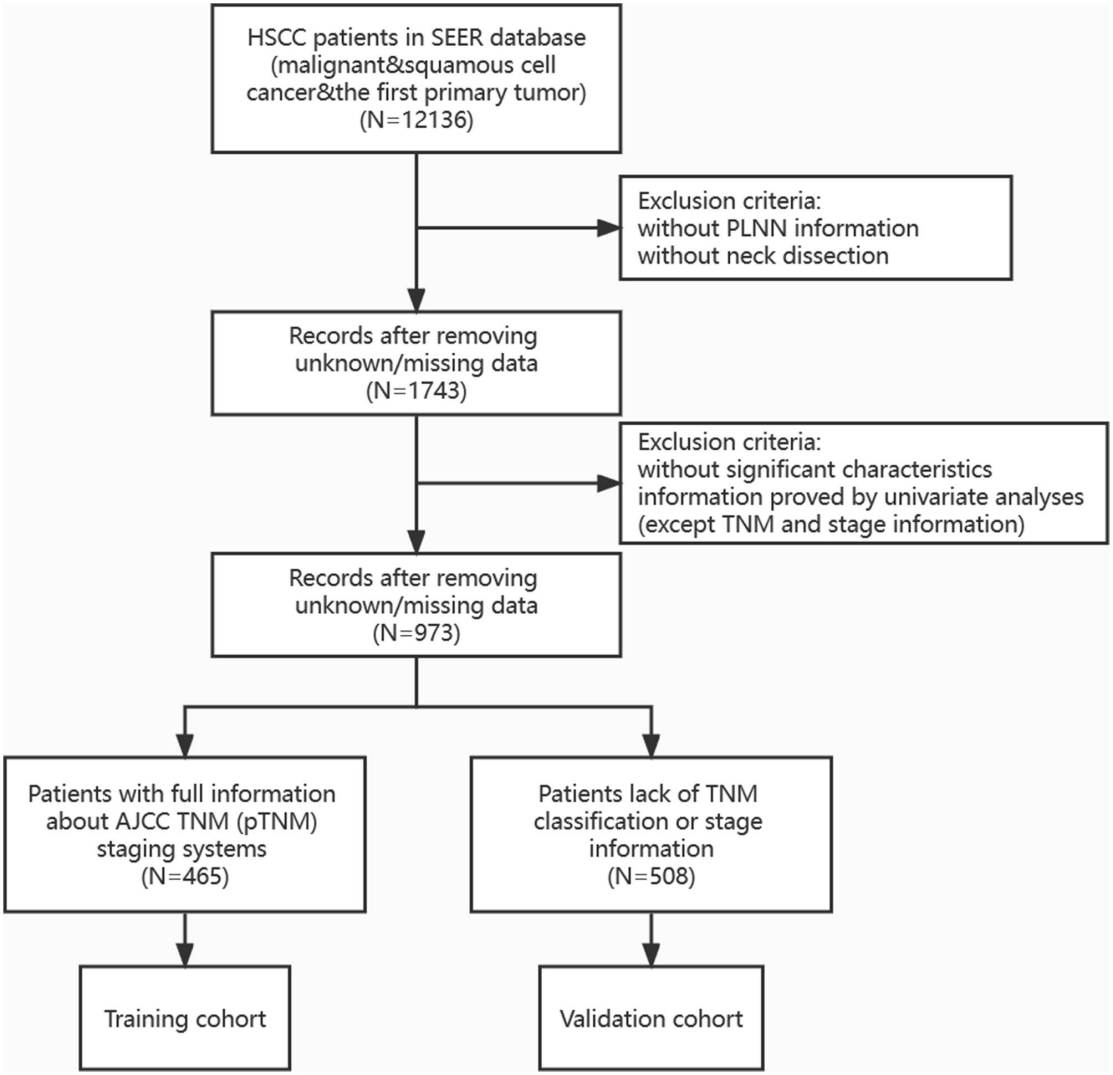
Flow diagram summarizing the process of inclusion and exclusion.

Pathologic diagnosis of HPSCC was based on the primary site using the International Classification of Diseases for Oncology, third edition, including the codes C129, C130, C131, C132, C138, and C139. Patients with the first primary cancer of HPSCC in the SEER database between 2004 and 2015 were identified (*n* = 12,136, Figure 1). The inclusion criteria were as follows: (1) histology of squamous cell carcinoma, including malignancy and carcinoma *in situ*, (2) HPSCC was reported as the first primary tumor, and (3) patients underwent neck dissection (8). Patients lacking the number of regional LNs or lacking complete information on clinical and pathological characteristics (such as primary site, marital status, age, chemotherapy, and radiation sequence with surgery, which were significant survival predictors proved by univariate analyses) were excluded from this study. After the exclusion of ineligible patients, the remaining HPSCC cases (*n* = 973) were divided into two cohorts: (1) training cohort, consisting of patients with complete TNM staging data (*n* = 465); (2) validation cohort, consisting of the remaining 508 patients without TNM stage information, to verify the prognostic ability of the training model.

### Patient Characteristics

Basic demographic information such as gender, age, marital status, tumor primary site, T classification, N classification, M classification, cancer stage, tumor grade, and treatment modalities was considered. PLNN was recorded as the exact number of regional nodes examined by a pathologist, who confirmed tumor infiltration. Owing to the similarity of the sixth and seventh AJCC cancer staging systems for patients with HPSCC in the SEER database, we applied both editions to define the TNM classifications and cancer stages (the sixth and seventh AJCC editions included patients from 2004 to 2009 and 2010 to 2015, respectively).

### Statistical Analyses

Continuous variables were presented as the median and maximum/minimum value and categorical variables were presented as frequencies and percentages. We conducted univariate Cox regression analyses using the Wald test to identify the confounding variables. The backward selection algorithm was used for model selection, and non-significant factors were excluded from further multivariable analyses. Multivariable Cox proportional hazards regression analyses were conducted to calculate the hazard ratio (HR) and 95% CI (Confidence Interval), which were used to analyze whether the PLNN could serve as a potential factor in predicting overall survival (OS) and cause-specific survival (CSS). OS was defined from the date of diagnosis to the date of death or last follow-up, with death as an event. Due to the fact that cancer patients may die from complications or other unexpected reasons, we applied CSS as the survival outcome which was defined as the date of diagnosis to the date of death due to HPSCC.

The cut-offs for PLNN were determined by comparing the HR of exact positive node numbers (1, 2, 3, 4, 5, 6, 7, and >7) after adjusting for gender, age, race, and marital status. Six models were built based on different combinations of PLNNs, cancer stages, and T, N, and M classifications. The concordance index (C-index) was processed to evaluate and compare the performance of each model with a value ranging from 0 to 1, where 0.5 corresponds to random chance and 1 corresponds to perfect discriminative ability.

All analyses were performed using R 3.6.3 (R Development Core Team, Vienna, Austria), and *P* < 0.05 were considered statistically significant.

## Results

### Patient Characteristics and Significant Prognostic Factors

Characteristics of patients with HPSCC after the exclusion, including the training cohort (*n* = 465) and validation cohort (*n* = 508), were summarized in Table 1 and Supplementary Table 1.

**Table 1:** Clinical and demographic characteristics of total patients (full samples, *N* = 973).

**Characteristic**	**Training cohort**				**Validation cohort**				**Global**
	**Total**	**PLNN (*n* = 0)**	**PLNN (1 < *n* ≤5)**	**PLNN (*n* > 5)**	***p*-Value**	**Total**	**PLNN (*n* = 0)**	**PLNN (1 < *n* ≤5)**	**PLNN (*n* > 5)**	***p*-Value**	***p*-Value**
	**(*N* = 465)**	**(*N* = 88)**	**(*N* = 285)**	**(*N* = 92)**		**(*N* = 508)**	**(*N* = 83)**	**(*N* = 319)**	**(*N* = 106)**		
**Primary site**											
Pyriform sinus	60%	53 %	61%	63%	0.320	68%	60%	69%	69%	0.44	**0.003**
Post-cricoid region	2%	3%	2%	1%		2%	1%	2%	4%		
Aryepiglottic fold	6%	10%	6%	3%		4%	5%	3%	3%		
Posterior wall	3%	3%	4%	2 %		6%	7%	5%	8%		
Overlapping lesion	4%	3%	2%	8%		3%	2%	3%	5%		
Hypopharynx, NOS	25%	26%	25%	23%		17%	24%	17%	11%		
**Sex**											
Female/Male	15/85%	17/83%	14/86%	13/87%	0.740	17/83%	17/83%	18/82%	15/85%	0.84	0.370
**Race**											
Black	13%	12%	10%	22%		17%	20%	15%	20%		
Others	6%	5%	6%	7%	0.065	8%	6%	9%	8%	0.67	**0.042**
White	81%	83%	84%	72%		75%	73%	76%	72%		
**Age**											
Mean (sd)	61.2 (9.7)	62.5 (10.9)	60.3 (9.2)	62.7 (9.9)	0.063	61.3 (10.3)	61.5 (10.8)	61.3 (10.2)	61.4 (10.4)	0.94	0.790
Median (Min, Max)	60 (31, 87)	62 (31, 86)	60 (39, 87)	62 (40, 86)		61 (29, 89)	61 (30, 89)	60 (29, 86)	61 (41, 85)		
**Marital status**											
Married	50%	62%	49%	39%	**0.021**	50%	57%	49%	49%	0.44	0.360
Single/Others	26/24%	24/14%	25/25%	30/30%		23%/27%	24/19%	22/29%	25/26%		
**Surgery primary site**											
No	20%	19%	23%	10%	**0.011**	22%	19%	24%	18%	0.62	0.150
Local	14%	15%	14%	11%		9%	7%	11%	7%		
Pharyngectomy	13%	17%	13%	9%		16%	18%	15%	15%		
PWM*	45%	45%	41%	55%		43%	43%	41%	47%		
Radical Pharyngectomy	9%	3%	8%	15%		10%	12%	9%	13%		
**Chemotherapy**											
No/Unknown	40%	56%	39%	27%	**<0.001**	67%	72%	66%	63%	0.42	**<0.001**
Yes	60%	44%	0%	73%		33%	28%	34%	37%		
**Radiation sequence with surgery**											
After	67%	40%	73%	72%	**<0.001**	68%	52%	69%	78%	0.012	**0.034**
Before	12%	24%	11%	4%		8%	11%	8%	4%		
Both	2%	2%	2%	2%		4%	5%	4%	3%		
No radiation	19%	34%	14%	22%		20%	33%	19%	15%		
**T**											
T1/T2	14/26%	15/33%	16/25%	9/23%	0.150						
T3/T4	17/43%	18/34%	17/42%	14/54%							
***N***											
N0/N1	15/18%	80/7%	0/27%	0/1%	**<0.001**						
N2/N3	61/6%	8/6%	67/6%	91/8%							
**M**											
M0/M1	98/2%	100/0%	100/0%	93/7%	**<0.001**						
**Stage**											
I, II	7%	35%	0%	0%	**<0.001**						
III	11%	20%	12%	0%							
IV	82%	44%	88%	100%							

Including training cohort (N = 465) and validation cohort (N = 508). PWM^*^ Pharyngectomy with mandibulectomy (marginal, segmental, hemi-, and/or laryngectomy). Radiation sequence refers to radiation sequence with surgery. Bold value indicated that P < 0.05 were considered statistically significant.

In univariate analyses (Supplementary Table 2), patients who had more advanced TNM stages or more PLNN, with tumor size >2 cm or primary tumor site located in overlapping lesions of hypopharynx had a poor prognosis (HR > 1, *p* < 0.01). The primary site, age, marital status, the primary site of surgery, chemotherapy, and radiation/surgery sequence were further selected (backward selection) for multivariable Cox regression analyses.

### Patient Characteristics and Prognostic Values of Clinical Factors by PLNN Cut-Offs

**Figure 2:**
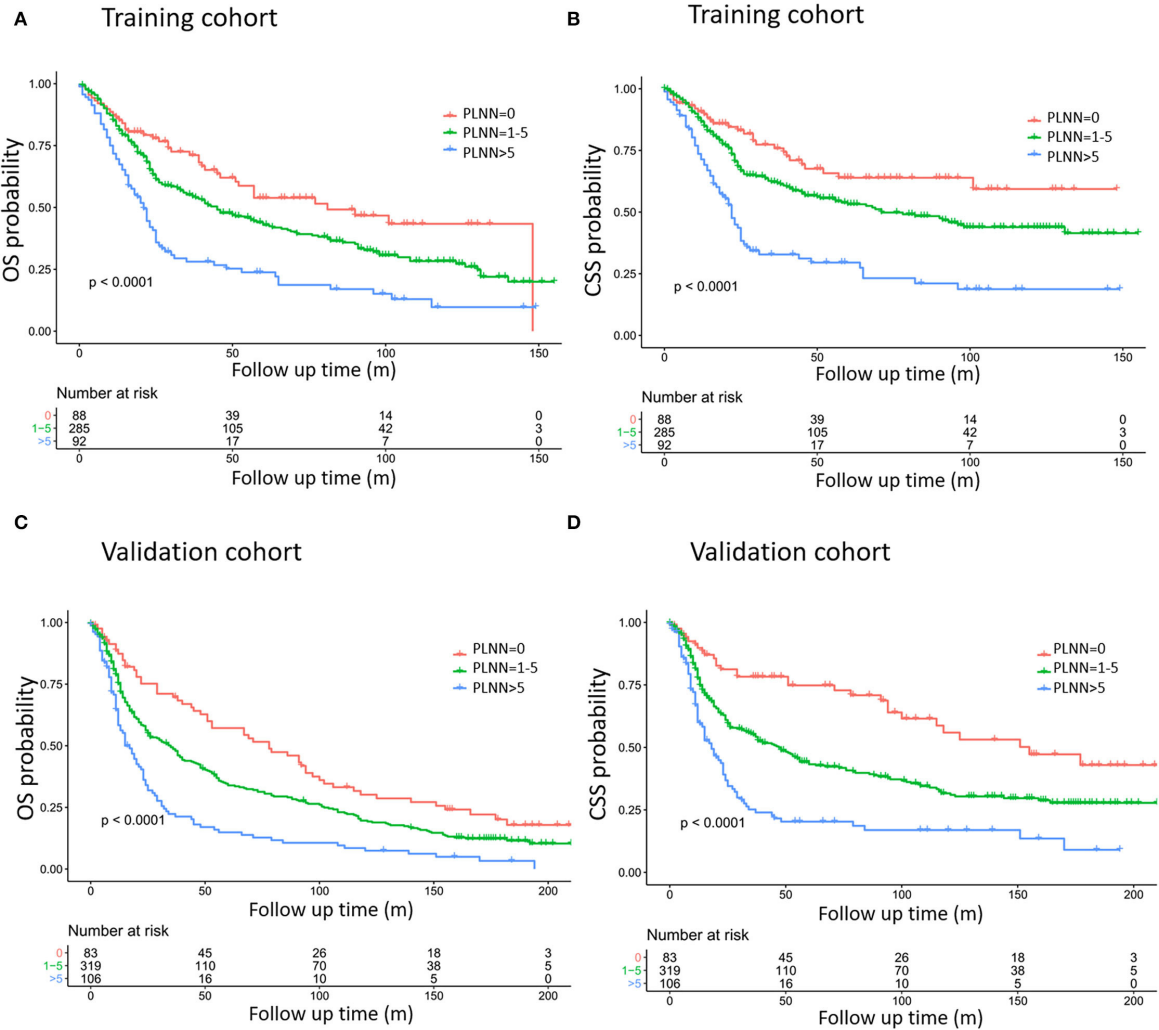
Kaplan–Meier curves estimating overall survival (OS) **(A,C)** and cause-specific survival (CSS) **(B,D)** based on training cohort **(A,B)** and validation cohort **(C,D)** definition of training cohort and validation cohort referred to Table 1. The exact data of survival rates are referred to in Table 2.

Our multivariate analysis revealed that increased PLNN was independently associated with decreased OS and CSS (Table 2). When the PLNN increased from 5 to 6, the HR of OS dramatically increased from 1.14 to 2.34 (95% CI: 1.75–3.14), therefore, we divided PLNN into three groups (PLNN 0, PLNN 1–5, and PLNN > 5) for further analysis. The survival curves of patients in the PLNN 0, PLNN 1–5, and PLNN > 5 groups showed significant differences, demonstrating the good discriminatory ability of the PLNN cut-offs (Figure 2, Supplementary Table 3), and consistent trends were also observed in the subgroups of age and gender (Supplementary Figure 1). Compared to HPSCC patients without LN metastases, patients whose PLNNs were between one and five (PLNN 1–5, HR for OS: 1.27, 95% CI: 1.09–1.47, *p* = 0.002; HR for CSS: 1.46, 95% CI: 1.2–1.78, *p* < 0.001) and PLNNs were over five (PLNN > 5, HR for OS: 2.29; 95% CI: 1.92–2.74, *p* < 0.001; HR for CSS: 3, 95% CI: 2.41–3.75, *p* < 0.001) had a higher risk of death. Patient characteristics shown by different PLNN cut-offs are presented in Supplementary Table 1.

**Table 2:** Multivariable analysis for the increasing positive lymph nodes numbers and the cut-offs.

**Positive lymph nodes numbers**	**OS**		**CSS**	
	**HR (95% CI)**	***P*-Value**	**HR (95% CI)**	***P*-Value**
0	Reference		Reference	
1*	**1.07 (0.89–1.28)**	**0.478**	**1.09 (0.86–1.39)**	**0.467**
2	1.22 (1.01–1.47)	0.035	1.37 (1.08–1.75)	0.010
3	1.47 (1.2–1.79)	<0.001	1.86 (1.45–2.38)	<0.001
4	1.73 (1.36–2.21)	<0.001	2.09 (1.55–2.82)	<0.001
5*	**1.44 (1.12–1.86)**	**0.005**	**1.83 (1.34–2.5)**	**<0.001**
6	2.34 (1.75–3.14)	<0.001	3.28 (2.34–4.6)	<0.001
7	2.33 (1.7–3.19)	<0.001	2.69 (1.85–3.92)	<0.001
>7	2.28 (1.87–2.78)	<0.001	3.02 (2.38–3.85)	<0.001
**Cut-Off value of 1 and 5**				
**The cut-offs for positive lymph nodes numbers**	**OS**		**CSS**	
	**HR (95% CI)**	***P*** **-Value**	**HR (95% CI)**	***P*** **-Value**
0	Reference		Reference	
1–5	1.27 (1.09–1.47)	0.002	1.46 (1.2–1.78)	<0.001
>5	2.29 (1.92–2.74)	<0.001	3 (2.41–3.75)	<0.001

Adjusted by primary site, sex, age, race, marital status. Multivariable analysis revealed that the increased PLNN were independently associated with the decreased OS and CSS. The HR of OS was dramatically increased to 2.34 (95% CI: 1.75 ~ 3.14) when the PLNN reached to 6. Bold value emphasized the reason why choose 1 and 5 as cut-off values. *Emphasized the cut-off value we finally chose (1 and 5).

### Survival Predicting Models Based on PLNN and TNM Staging System

We further conducted six Cox regression models based on different combinations of PLNN (with cut-off values of 1 and 5), T classification (T1–T4), N classification (N0–N3), M classification (M0–M1), and cancer stage (Stage I–IV) to predict survival outcomes. Model 1 (PLNN), model 2 (cancer stages), and model 3 (TNM) were established solely based on PLNN, cancer stage, and TNM classification, respectively. Model 4 (PLNN + cancer stages) was developed by integrating PLNN and cancer stages; model 5 (PLNN + T + M) incorporated PLNN, T, and M classifications, and model 6 (PLNN + TNM) combined PLNN with TNM classification.

As shown in Table 3, the model of PLNN (model 1, C-index for OS:0.664; C-index for CSS:0.677) showed a comparable prognostic effect when compared with the models utilizing cancer stages (model 2, C-index for OS:0.658; C-index for CSS:0.672) or TNM classification (model 3, C-index for OS:0.674; C-index for CSS:0.691), indicating that PLNN was a reasonable prognostic factor. Additionally, the prognostic prediction effects of PLNN gradually improved when incorporating with cancer stages (model 4, C-index for OS:0.672; C-index for CSS:0.693), TM classifications (model 5, C-index for OS:0.681; C-index for CSS:0.697), or TNM classifications (model 6, C-index for OS:0.682; C-index for CSS:0.702), suggesting that PLNN could serve as a surrogate supplement for TNM classification and cancer stage.

**Table 3:** Comparison of six survival predicting models based on different permutations and combinations of PLNN (with the cut-off value of 1 and 5), AJCC T- (T1–T4), N- (N0–N3), M- (M0–M1) classification and AJCC cancer stage systems (Stage I–IV).

**Models**			**OS**			**CSS**		
			**HR (95% CI)**	***P*-Value**	**C-Index**	**HR (95% CI)**	***P*-Value**	**C-Index**
	Model 1: PLNN	0	Reference		0.664	Reference		0.677
	1–5	1.9 (1.32–2.76)	0.001		2.03 (1.3–3.16)	0.002	
	>5	3.32 (2.18–5.05)	<0.001		4.31 (2.64–7.03)	<0.001	
	Model 2: Stage	I/II	Reference		0.658	Reference		0.672
	III	2.34 (1.13–4.86)	0.023		2.31 (0.8–6.63)	0.121	
	IV	3.68 (1.9–7.12)	<0.001		6.15 (2.4–15.77)	<0.001	
Model 3: TNM classification	T classification	T1	Reference		0.674	Reference		0.691
		T2	1.76 (1.1–2.82)	0.019		1.74 (0.99–3.04)	0.053	
		T3	2.24 (1.32–3.8)	0.003		2.23 (1.2–4.13)	0.011	
		T4	2.63 (1.61–4.31)	<0.001		3.27 (1.83–5.84)	<0.001	
	N classification	N0	Reference			Reference		
		N1	1.58 (1–2.5)	0.052		1.63 (0.92–2.91)	0.096	
		N2	2.14 (1.42–3.2)	<0.001		2.69 (1.63–4.45)	<0.001	
		N3	2.96 (1.53–5.72)	0.001		4.1 (1.93–8.7)	<0.001	
	M classification	M0	Reference			Reference		
		M1	2.64 (1.15–6.09)	0.023		1.49 (0.47–4.76)	0.501	
Model 4: PLNN stage	PLNN	0	Reference		0.672	Reference		0.693
		1–5	1.45 (0.96–2.19)	0.079		1.36 (0.84–2.18)	0.211	
		>5	2.42 (1.51–3.88)	<0.001		2.64 (1.56–4.48)	<0.001	
	Stage	I/II	Reference			Reference		
		III	1.77 (0.81–3.89)	0.155		1.83 (0.61–5.5)	0.283	
		IV	2.33 (1.1–4.97)	0.028		3.94 (1.41–11.03)	0.009	
Model 5: PLNN TM classification	T classification	T1	Reference		0.681	Reference		0.697
		T2	1.76 (1.09–2.84)	0.020		1.73 (0.98–3.04)	0.059	
		T3	2.37 (1.39–4.05)	0.002		2.41 (1.29–4.5)	0.006	
		T4	2.5 (1.53–4.11)	<0.001		3.01 (1.68–5.38)	<0.001	
	PLNN	0	Reference			Reference		
		1–5	1.86 (1.29–2.67)	0.001		1.95 (1.26–3.03)	0.003	
		>5	3.07 (2.01–4.68)	<0.001		4.09 (2.51–6.66)	<0.001	
	M classification	M0	Reference			Reference		
		M1	2.06 (0.88–4.81)	0.096		1.05 (0.33–3.4)	0.930	
Model 6: PLNN TNM classification	T classification	T1	Reference		0.682	Reference		0.702
		T2	1.85 (1.14–2.99)	0.012		1.79 (1.02–3.17)	0.044	
		T3	2.34 (1.37–4)	0.002		2.34 (1.25–4.36)	0.008	
		T4	2.64 (1.6–4.35)	<0.001		3.21 (1.79–5.76)	<0.001	
	N classification	N0	Reference			Reference		
		N1	0.67 (0.27–1.66)	0.386		0.82 (0.31–2.16)	0.682	
		N2	0.76 (0.3–1.89)	0.550		1.05 (0.4–2.77)	0.923	
		N3	1.22 (0.46–3.22)	0.694		1.82 (0.64–5.12)	0.260	
	M classification	M0	Reference			Reference		
		M1	1.98 (0.85–4.65)	0.115		1.03 (0.32–3.33)	0.961	
	PLNN	0	Reference			Reference		
		1–5	2.45 (1.08–5.57)	0.032		2.06 (0.9–4.76)	0.089	
		>5	3.88 (1.63–9.25)	0.002		3.97 (1.63–9.64)	0.002	

*i. Model 1 was established solely on the basis of PLNN; ii. Model 2 was based on AJCC cancer stage; iii. Model 3 was proposed by both PLNN and AJCC stage system; iv. Model 4 relied on the strength of AJCC TNM classification; v. Model 5 incorporated PLNN, AJCC T-, and M- classification; and vi. Model 6 utilized PLNN combined with AJCC TNM classification. Adjusted by primary site, marital status, age, chemotherapy, radiation sequence with surgery, and primary surgery site*.

### Prognostic Prediction Effects Verified by the Validation Cohort

We further conducted two subgroup analyses for survival outcomes by stratifying age and gender (Supplementary Figure 1). The generalizability of the survival prediction model was examined using a validation cohort. All models were adjusted for the primary site, marital status, age, chemotherapy, radiation/surgery sequence, and primary surgery site.

Consistent prognostic prediction performances of the models were observed in the validation cohort (C-index for OS, 0.638; C-index for CSS, 0.656; Table 4). As shown in Table 1, although some of the clinical factors demonstrated statistical differences between the training and validation cohorts, the overall model performance of the validation cohort was consistent with that of the training cohort (C-index: training cohort:0.664 for OS and 0.677 for CSS; validation cohort:0.638 for OS and 0.656 for CSS).

**Table 4:** Comparison of survival predicting model based on training cohort (*N* = 461) and validation cohort (*N* = 365).

**Models**	**OS**			**CSS**		
		**HR (95% CI)**	***P*-Value**	**C-Index**	**HR (95% CI)**	***P*-Value**	**C-Index**
Training cohort	0	Reference		0.664	Reference		0.677
	1–5	1.9 (1.32–2.76)	0.001		2.03 (1.3–3.16)	0.002	
	>5	3.32 (2.18–5.05)	<0.001		4.31 (2.64–7.03)	<0.001	
Validation cohort	0	Reference		0.638	Reference		0.656
	1–5	1.57 (1.17–2.11)	0.003		2.17 (1.46–3.22)	<0.001	
	>5	2.98 (2.11–4.19)	<0.001		4.31 (2.78–6.68)	<0.001	

*Definition of training cohort model is the same as Model 1 in Table 3 (only based on PLNN). Adjusted by primary site, marital status, age, chemotherapy, radiation sequence with surgery, and primary surgery site*.

## Discussion

Our current study showed the prognostic effects of PLNNs on patients with HPSCC. In continuous multivariable regression models, we observed that successive increasing PLNNs were associated with an increased risk of death, and when patients were classified by PLNN (PLNN 0; PLNN 1–5; PLNN > 5), significant prognostic differences were observed in both the training and validation cohorts. We further confirmed that PLNN was a superior supplement to enhance the prognostic prediction value of the current 6th and 7th AJCC staging system.

Due to early and diffuse submucosal infiltration along with an extensive lymphatic network, HPSCC has a higher rate of LN metastasis than other types of HNSCC, where more than 50% of patients present with positive LNs (9). It has been widely accepted that metastatic LNs are independent prognostic factors for the survival of HNSCC (10). The concept of LN ratio (LNR) or LN density was calculated as the number of positive LNs divided by the number of LNs harvested from neck dissection (11, 12). A recent meta-analysis (13) demonstrated that LNR is an important CSS predictor for patients with HPSCC along with LN metastasis. Retrospective studies demonstrated that patients with hypopharyngeal cancer had a high risk of retropharyngeal LN involvement, and frequently progressed to distant metastasis with dismal outcomes (14, 15).

Some previous studies (16–19) suggested that the prognostic value of PLNN could even surpass other clinical factors such as tumor size and the laterality of LN. However, the prognostic effect of PLNN on patients with HPSCC remains poorly understood. Despite the comprehensiveness of the AJCC TNM staging system that has considered the diameter and location of LNs and distant metastasis information, it did not consider the value of PLNN, which could provide a simpler, more precise, and reliable prognosis reference.

Besides, our results showed that the performance of the model 1–5, except for the model 6 (PLNN + TNM), was not excellent (c-index < 0.7). Consistently, previous studies showed similar inferior prognostic effects of 6th and 7th TNM staging systems on patients with hypopharyngeal cancer, indicating that a supplement for TNM staging system for patients with HPSCC is still necessary.

Although our study showed potential prognostic effects of PLNN on patients with HPSCC, a few limitations still exist. First, the SEER database has limited clinical and treatment information, which restricts our access to relevant data for clinical LN staging *via* ultrasound or computed tomography, extracapsular spread, and the method and dose of adjuvant radiotherapy. Second, the results of PLNN might be affected by the neck dissection and collection performed by surgeons and the LN examination performed by pathologists. In addition, the SEER database (2004–2015) did not include staging information of the latest AJCC 8th edition. Our study only considered that PLNN can be used as a supplement to help improve the prognostic evaluation effect of the AJCC 6th and 7th staging strategy, while it still needs to be further verified in the HPSCC cohort which was staged by the 8th AJCC edition in the future. Despite these concerns, PLNN is suggested to quantify LN metastasis, which is easier to apply in clinical practice and has the potential to help predict HPSCC prognosis.

## Conclusions

Positive lymph node number was a potential prognostic factor associated with OS and CSS in patients with HPSCC. The survival model incorporating PLNN and TNM classification performed better than other models that are based on any single variable. PLNN may serve as a survival predictor for patients with HPSCC and a supplement to enhance the evaluation results of TNM cancer staging systems.

## Data Availability Statement

The original contributions presented in the study are included in the article/Supplementary Material, further inquiries can be directed to the corresponding author/s.

## Ethics Statement

The studies involving human participants were reviewed and approved by West China Hospital review board. Written informed consent for participation was not required for this study in accordance with the national legislation and the institutional requirements.

## Author Contributions

FC and WP designed the study. DC and YR analyzed and interpreted data in the included studies. MM and KQ performed quality control of data and algorithms. YL and JLi performed the statistical analysis. YD contributed to the acquisition of data. JZ helped perform the analysis with constructive discussions. WP, YL, and JLi were major contributors for writing the manuscript. FC and HW reviewed the manuscript. All authors read and approved the final manuscript.

## Conflict of Interest

The authors declare that the research was conducted in the absence of any commercial or financial relationships that could be construed as a potential conflict of interest.

## Publisher's Note

All claims expressed in this article are solely those of the authors and do not necessarily represent those of their affiliated organizations, or those of the publisher, the editors and the reviewers. Any product that may be evaluated in this article, or claim that may be made by its manufacturer, is not guaranteed or endorsed by the publisher.

## Abbreviations

SEER: database, the Surveillance, Epidemiology and End Results database
PLNN: positive lymph node number
AJCC: the American Joint Committee on Cancer
TNM: staging system, tumor, lymph node and metastasis staging system
HPSCC: hypopharyngeal squamous cell carcinoma
OS: overall survival
CSS: cancer-specific survival
LNM: lymph nodes metastasis
HR: hazard ratio
CI: confidence interval
LNs: lymph nodes
LNR: lymph nodes ratio.
