# Supplementary material for: The Prognostic Prediction Value of Positive Lymph Nodes Numbers for the Hypopharyngeal Squamous Cell Carcinoma
Source: Front Med (Lausanne). 2022 Jul 4;9:898483. doi: 10.3389/fmed.2022.898483 (PMC9291443; doi:10.3389/fmed.2022.898483)
Supplement: Supplementary file 1 [file Data_Sheet_1.DOCX]

***Table. S1 The overall patient characteristics by different PLNN cut-offs (N=1743)***

| **Covariate** | **Total** | **PLNN(n=0)** | **PLNN(1<n≤5)** | **PLNN(n>5)** | **p-value** |
| --- | --- | --- | --- | --- | --- |
|  | **(N=1743)** | **(N=300)** | **(N=1107)** | **(N=336)** |  |
| **Primary Site** |  |  |  |  |  |
| Pyriform sinus | 71% | 64% | 73% | 71% | **0.025** |
| Postcricoid region | 2% | 2% | 2% | 3% |  |
| Aryepiglottic fold | 4% | 6% | 4% | 3% |  |
| Posterior wall | 4% | 5% | 4% | 4% |  |
| Overlapping lesion | 3% | 2% | 2% | 4% |  |
| Hypopharynx, NOS | 16% | 20% | 15% | 15% |  |
| **Sex** |  |  |  |  |  |
| Female/Male | 17%/83% | 18%/82% | 18%/82% | 15%/85% | 0.470 |
| **Age** |  |  |  |  |  |
| Mean (sd) | 61.2 (10) | 61.6 (10.7) | 61.1 (9.6) | 61.2 (10.6) | 0.490 |
| Median (Min,Max) | 61 (25,93) | 62 (25,89) | 61 (29,93) | 61 (37,87) |  |
| **Race** |  |  |  |  |  |
| Black/Others | 17%/7% | 18%/8% | 15%/7% | 23%/7% | **0.017** |
| White | 76% | 75% | 78% | 71% |  |
| **Marital Status** |  |  |  |  |  |
| Married | 53% | 57% | 53% | 48% | 0.210 |
| Single/Others | 20%/27% | 20%/23% | 20%/27% | 23%/29% |  |
| Missing | 58 | 6 | 38 | 14 |  |
| **Tumor Size** |  |  |  |  |  |
| ≤2/>2 | 20%/80% | 21%/79% | 21%/79% | 13%/87% | 0.020 |
| Missing | 388 | 62 | 242 | 84 |  |
| **Surgery Primary Site** |  |  |  |  |  |
| No | 21% | 19% | 23% | 14% | 0.008 |
| Local | 11% | 11% | 12% | 8% |  |
| Pharyngectomy | 14% | 17% | 14% | 12% |  |
| PWM* | 44% | 46% | 41% | 50% |  |
| Radical Pharyngectomy | 10% | 7% | 8% | 15% |  |
| Missing | 739 | 125 | 482 | 132 |  |
| **Radiotherapy** |  |  |  |  |  |
| None, Unknown | 19% | 30% | 16% | 15% | <0.001 |
| Yes | 81% | 70% | 84% | 85% |  |
| **Extension** |  |  |  |  |  |
| Local | 73% | 73% | 72% | 73% | 0.230 |
| Metastasis | 2% | 1% | 2% | 4% |  |
| Regional | 25% | 25% | 26% | 23% |  |
| Missing | 116 | 18 | 74 | 24 |  |
| **Radiation sequence with surgery** |  |  |  |  |  |
| After | 72% | 55% | 74% | 80% | **<0.001** |
| Before | 7% | 12% | 7% | 2% |  |
| Both | 2% | 2% | 2% | 2% |  |
| No radiation | 19% | 30% | 17% | 16% |  |
| Missing | 3 | 0 | 1 | 2 |  |
| [**Chemotherapy**](javascript:;) |  |  |  |  |  |
| No/Unknown | 69%  31% | 76% | 70% | 61% | **<0.001** |
| Yes | 31% | 24% | 30% | 39% |  |
| **T** |  |  |  |  |  |
| T1/T2 | 14%/26% | 14%/31% | 16%/26% | 8%/26% | 0.150 |
| T3/T4 | 17%/43% | 20%/35% | 16%/42% | 15%/54% |  |
| Missing | 1250 | 208 | 806 | 236 |  |
| **N** |  |  |  |  |  |
| N0/N1 | 14%/19% | 78%/7% | 0%/27% | 0%/3% | **<0.001** |
| N2/N3 | 60%/7% | 9%/5% | 67%/6% | 87%/10% |  |
| Missing | 1215 | 205 | 781 | 229 |  |
| **M** |  |  |  |  |  |
| M0/M1 | 98%/2% | 100%/0% | 99%/1% | 94%/6% | **0.002** |
| Missing | 1215 | 204 | 782 | 229 |  |
| **Stage** |  |  |  |  |  |
| I, II | 6% | 33% | 0% | 0% | **<0.001** |
| III/IV | 11%/83% | 21%/46% | 12%/88% | 0%/100% |  |
| Missing | 1223 | 206 | 788 | 229 |  |
| **Grade** |  |  |  |  |  |
| I/II | 4%/49% | 6%/52% | 5%/49% | 3%/45% | 0.087 |
| III/IV | 45%/2% | 41%/1% | 44%/2% | 50%/2% |  |
| Missing | 144 | 31 | 94 | 19 |  |

***Table. S2 Univariable analysis for overall survival and cause-specific survival survival by clinico-demographic variables.*** *Radiation sequence refers to radiation sequence with surgery.*

| **Covariate** | **Categories** | **OS** | | **Global p-value** | **CSS** | | **Global p-value** |
| --- | --- | --- | --- | --- | --- | --- | --- |
|  |  | **HR (95% CI)** | **p-value** |  | **HR (95% CI)** | **p-value** |  |
| **Primary Site** | Pyriform sinus | Reference |  | 0.017 | Reference |  | 0.003 |
|  | Postcricoid region | 0.97 (0.66,1.42) | 0.86 |  | 1.02 (0.65,1.59) | 0.93 |  |
|  | Aryepiglottic fold, hypopharyngeal | 0.88 (0.66,1.19) | 0.42 |  | 0.77 (0.52,1.13) | 0.18 |  |
|  | Posterior wall of hypopharynx | 1.12 (0.86,1.47) | 0.4 |  | 1.27 (0.93,1.73) | 0.14 |  |
|  | Overlapping lesion of hypopharynx | 1.6 (1.15,2.21) | 0.0047 |  | 1.8 (1.25,2.58) | 0.0014 |  |
|  | Hypopharynx, NOS | 0.87 (0.74,1.01) | 0.061 |  | 0.86 (0.72,1.04) | 0.11 |  |
| **Sex** | Female | Reference |  | 0.490 | Reference |  | 0.750 |
|  | Male | 0.95 (0.83,1.09) | 0.49 |  | 0.97 (0.82,1.15) | 0.75 |  |
| **Race** | Black | Reference |  | $<$0.001 | Reference |  | $<$0.001 |
|  | Others | 0.78 (0.61,0.98) | 0.033 |  | 0.82 (0.63,1.07) | 0.15 |  |
|  | White | 0.71 (0.62,0.81) | $<$0.001 |  | 0.66 (0.56,0.78) | $<$0.001 |  |
| **Marital Status** | Married | Reference |  | $<$0.001 | Reference |  | $<$0.001 |
|  | Other | 1.28 (1.13,1.45) | $<$0.001 |  | 1.28 (1.1,1.49) | 0.0014 |  |
|  | Single | 1.32 (1.15,1.52) | $<$0.001 |  | 1.42 (1.2,1.68) | $<$0.001 |  |
| **Age** | — | 1.02 (1.01,1.02) | $<$0.001 | $<$0.001 | 1.01 (1,1.02) | 0.0088 | 0.009 |
| **Grade** | I | Reference |  | 0.990 | Reference |  | 0.860 |
|  | II | 1.02 (0.78,1.34) | 0.89 |  | 0.97 (0.7,1.35) | 0.87 |  |
|  | III | 1.01 (0.77,1.33) | 0.92 |  | 1.01 (0.73,1.4) | 0.95 |  |
|  | IV | 0.95 (0.58,1.57) | 0.85 |  | 0.81 (0.43,1.55) | 0.53 |  |
| **Surgery Primary Site** | No | Reference |  | 0.001 | Reference |  | 0.010 |
|  | Local | 0.76 (0.57,1.02) | 0.067 |  | 0.69 (0.49,0.97) | 0.031 |  |
|  | Pharyngectomy | 0.95 (0.74,1.23) | 0.72 |  | 0.91 (0.67,1.22) | 0.52 |  |
|  | Pharyngectomy with mandi | 1.25 (1.03,1.52) | 0.026 |  | 1.17 (0.93,1.46) | 0.18 |  |
|  | Radical Pharyngectomy | 1.01 (0.76,1.35) | 0.94 |  | 0.92 (0.65,1.29) | 0.62 |  |
| **Radiation sequence** | After | Reference |  | $<$0.001 | Reference |  | 0.007 |
|  | Before | 0.76 (0.66,0.87) | $<$0.001 |  | 0.75 (0.64,0.88) | $<$0.001 |  |
|  | Both | 0.74 (0.58,0.93) | 0.011 |  | 0.76 (0.58,1.01) | 0.057 |  |
|  | No radiation | 0.82 (0.56,1.22) | 0.33 |  | 0.75 (0.46,1.23) | 0.26 |  |
| **Chemotherapy** | No/Unknown | Reference |  | <0.001 | Reference |  | 0.520 |
|  | Yes | 0.82 (0.72,0.92) | <0.001 |  | 0.95 (0.83,1.1) | 0.52 |  |
| **PLNN** | 0 | Reference |  | $<$0.001 | Reference |  | $<$0.001 |
|  | 1-5 | 1.23 (1.06,1.43) | 0.0057 |  | 1.42 (1.17,1.72) | $<$0.001 |  |
|  | $>$5 | 2.25 (1.89,2.68) | $<$0.001 |  | 3.01 (2.42,3.74) | $<$0.001 |  |
| **TumorSize** | ≤2 | Reference |  | <0.001 | Reference |  | $<$0.001 |
|  | >2 | 1.55 (1.32,1.81) | <0.001 |  | 1.8 (1.47,2.21) | <0.001 |  |
| **T- classification** | T1 | Reference |  | <0.001 | Reference |  | $<$0.001 |
|  | T2 | 1.95 (1.25,3.03) | 0.003 |  | 1.86 (1.11,3.12) | 0.019 |  |
|  | T3 | 2.18 (1.36,3.52) | 0.0013 |  | 2.06 (1.18,3.61) | 0.011 |  |
|  | T4 | 2.54 (1.67,3.85) | $<$0.001 |  | 2.75 (1.69,4.47) | $<$0.001 |  |
| **N- classification** | N0 | Reference |  | 0.022 | Reference |  | 0.002 |
|  | N1 | 1.22 (0.81,1.85) | 0.35 |  | 1.31 (0.78,2.21) | 0.3 |  |
|  | N2 | 1.6 (1.12,2.28) | 0.01 |  | 1.96 (1.26,3.07) | 0.003 |  |
|  | N3 | 1.82 (1.06,3.1) | 0.029 |  | 2.52 (1.38,4.63) | 0.0028 |  |
| **M- classification** | M0 | Reference |  | 0.0037 | Reference |  | 0.095 |
|  | M1 | 2.84 (1.4,5.75) | 0.0037 |  | 2.13 (0.88,5.19) | 0.095 |  |
| **Stage** | I/II | Reference |  | 0.0043 | Reference |  | $<$0.001 |
|  | III | 1.46 (0.73,2.91) | 0.28 |  | 1.54 (0.56,4.24) | 0.4 |  |
|  | IV | 2.22 (1.21,4.05) | 0.0096 |  | 3.84 (1.58,9.31) | 0.0029 |  |

***Table. S3 Three-year and five-year survival rates of HPSCC patients, by different number of positive LNs***

| **Overall Survival** | | | | | |  | **Cause-specific Survival** | | | | | |
| --- | --- | --- | --- | --- | --- | --- | --- | --- | --- | --- | --- | --- |
| **Strata** | **Event/Total** | **3 years** | | **5 years** | |  | **Strata** | **Event/Total** | **3 years** | | **5 years** | |
|  |  | **(95% CI)** | **p-value** | **(95% CI)** | **p-value** |  |  |  | **(95% CI)** | **p-value** | **(95% CI)** | **p-value** |
| total | 291/465 | 53%(48-58) | <0.001 | 41%(37-46) | <0.001 |  | total | 218/465 | 59%(54-64) | <0.001 | 51%(46-56) | <0.001 |
| 0 | 40/88 | 70%(61-80) | <0.001 | 54%(43-67) | <0.001 |  | 0 | 27/88 | 91%(85-97) | <0.001 | 64%(53-77) | <0.001 |
| 1~5 | 178/285 | 55%(50-62) | <0.001 | 43%(37-50) | <0.001 |  | 1~5 | 127/285 | 62%(57-69) | <0.001 | 53%(47-61) | <0.001 |
| >5 | 73/92 | 28%(19-42) | <0.001 | 24%(16-38) | <0.001 |  | >5 | 64/92 | 25%(18-36) | <0.001 | 20%(13-31) | <0.001 |


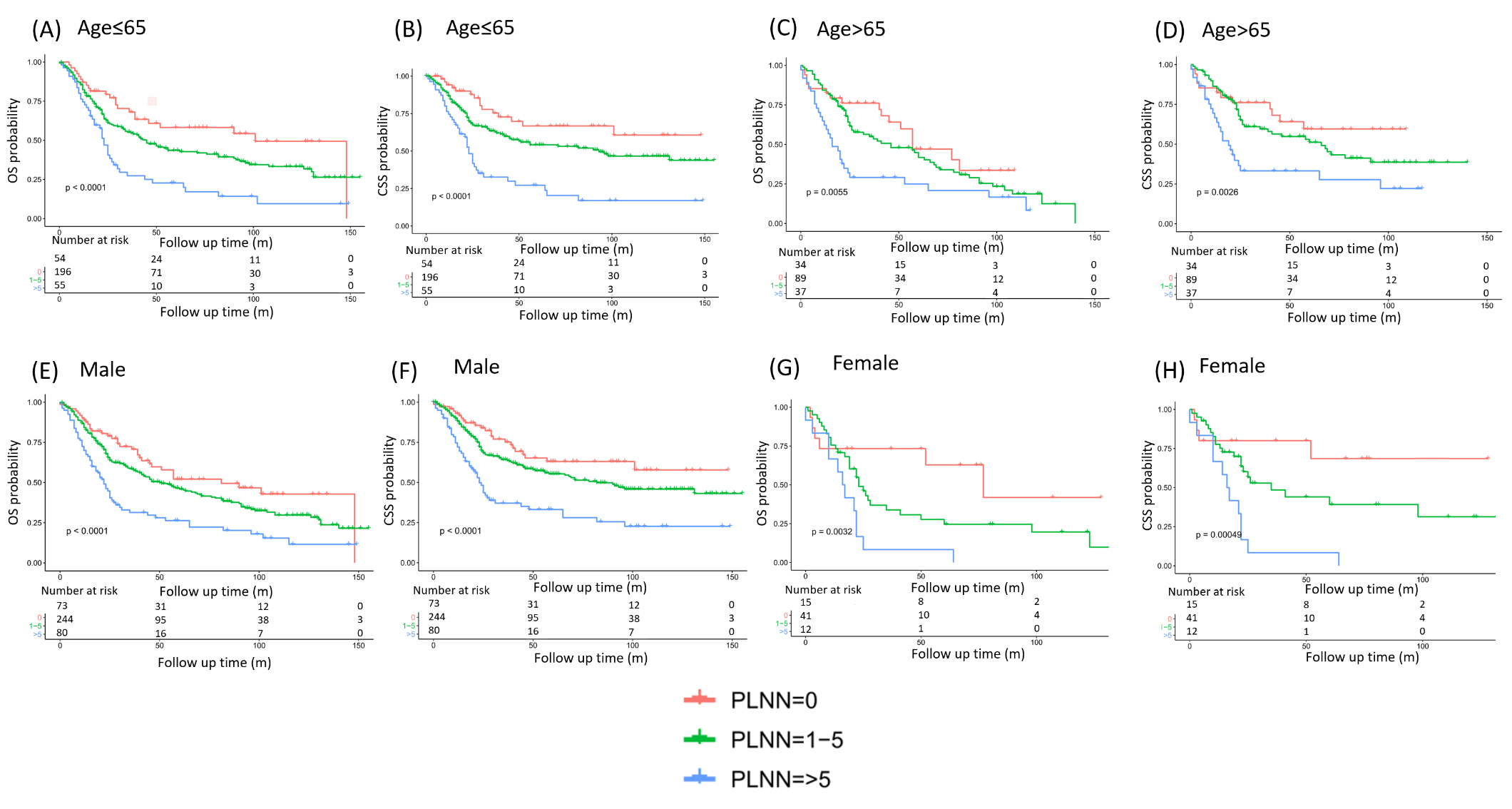


***Figure. S1 Kaplan-Meier curves estimating overall survival (OS) (ACEG) and cause-specific survival (CSS) (BDFH) based on subgroups.***

***(AB) Age≤65 (CD) Age＞65; (EF) Male (GH) Female***
